# Family planning awareness, utilization and associated factors among women of reproductive age attending psychiatric outpatient care, a cross- sectional study, Addis Ababa, Ethiopia

**DOI:** 10.1371/journal.pone.0238766

**Published:** 2020-09-04

**Authors:** Tigist Zerihun, Delayehu Bekele, Elizabeth Birhanu, Yoseph Worku, Negussie Deyesa, Markos Tesfaye

**Affiliations:** 1 Department of Psychiatry, St Paul’s Hospital Millennium Medical College, Addis Ababa, Ethiopia; 2 Department of Obstetrics and Gynecology, St Paul’s Hospital Millennium Medical College, Addis Ababa, Ethiopia; 3 Department of Public Health, St Paul’s Hospital Millennium Medical College, Addis Ababa, Ethiopia; 4 School of Public Health Addis Ababa University, Addis Ababa, Ethiopia; Ordu University, TURKEY

## Abstract

**Background:**

Women with mental illness have a special need for family planning as they carry a high risk of unplanned pregnancy, sexual violence and, the poor obstetric outcomes due to their mental illness, as well as teratogenicity from exposure to psychotropic medications lower antenatal care utilization.

**Objective:**

To assess knowledge, and utilization of family planning and associated factors among women attending psychiatric outpatient clinics in Addis Ababa.

**Methods:**

A cross-sectional study was conducted among 423 women attending the outpatient psychiatric clinics of three general and one specialized mental hospital in Addis Ababa, the capital city of Ethiopia. A structured and pretested questionnaire were administered by psychiatric nurses. Multiple logistic regression analysis was conducted to identify factors associated with utilization of family planning methods.

**Result:**

Four hundred twenty-two participants who had follow up at the psychiatric outpatient departments participated in the study. Almost 88% of participants had an unintended pregnancy. Only 68% of study participant had ever heard about Family planning. Just over one third (38.6%) reported current use of at least one method of Family planning. Of those not using family planning 73.3% had no intention to have children. And 38.8% did not have any intention to use Family Planning in the future. Fear of drug-interaction with psychiatric medication was the most common reason not to use contraceptives. Having one or two children was associated with higher utilization of family planning [adjusted odds ratio (95%, confidence interval) 2.05 (1.06, 3.99)].

**Conclusions:**

In this study, the majority of women with mental illness were not using family planning methods. The Awareness of the Family planning methods is lower than the national average. Education and counselling about family planning for women attending psychiatric outpatient departments should be strengthened.

## Introduction

Although family planning (FP) is essential for all women of reproductive age, it is particularly important for women with mental illness since they carry a high risk of unplanned pregnancy, vulnerability to sexual violence and poor obstetric outcome due to the mental illness as well as possible teratogenicity from exposure to some psychotropic medications and lower antenatal care utilization [1–6]. In addition, women with mental illnesses also need special consideration when using hormone- based contraceptives due to possible interactions with psychotropic medication although they can safely use other available methods [7].

Even though FP coverage is increasing worldwide, in the countries of sub Sharan Africa and other developing countries, the prevalence of contraceptive use remains low and the unmet need for FP services is high [8–11]. The situation in Ethiopia is similar [11, 12]: According to Ethiopian demographic health survey report, contraceptive coverage has increased from 6.3% in 2000 to 36% in 2019 among married women [13, 14]. Nevertheless, the unmet need is estimated to be more than 16% [15]. In the national survey of 2011, 25% of women surveyed did not want to have more children in the near future pregnancy. However, they were not using any form of contraceptives [16].

FP utilization is affected by many factors in low income settings, such as socio- cultural norms in which men dominate decision making because of the lower social status of women, education, residency, and income [17, 18].

Women with mental illness may face additional barriers due to poor access for health care, low levels of FP awareness, high levels of stigma and various disease related issues [19]. However, there is little information regarding FP awareness and utilization among women with mental illness in low income countries.

## Materials and methods

### Study design and setting

We employed a facility-based cross-sectional study design. We obtained ethical approval from the institutional review board of St Paul’s Hospital Millennium Medical College. The study was conducted in Addis Ababa (AA) City administration from September to December 2016. According to the 2007 census, Addis Ababa has a population of 2,687,593 people of which 34.8% are women of reproductive age and 28.4% are using contraception [1]. The data was collected from the outpatient psychiatric clinics of three general Hospitals (St. Paul’s Hospital, Yekatit 12 Hospital, and Zewditu Memorial Hospital), and one Psychiatric Hospital (Amanuel Mental Specialized Hospital). These hospitals deliver mental health services by psychiatrists or psychiatric residents.

### Sampling

#### Sample size determination

The sample size was calculated based on the following assumptions: proportion of patients who utilize FP service (𝑃 = 50%) taken to obtain the maximum sample size, 𝑍 = 1.96 at 95% confidence interval, 𝑑 = the level of precision (0.05), and nonresponse rate = 10%; this gave a total required sample size of n = 422.

#### Sampling procedure

In this study, we enrolled a total of 422 participants. The study was recruited consecutively from psychiatric outpatient clinics of the four Hospitals (three general and one psychiatric Hospitals). All consenting women aged 18–49 years were included who presented in the study period. Critically ill women and women who were unable to respond to the interviews were excluded after assessment by psychiatric nurses for their capacity to consent.

### Data collection methods and instrument

An interviewer-administered structured questionnaire was used to collect the data. The questionnaire was developed in English after reviewing similar studies carried out previously using multi-culturally validated tools [20, 21] with adaptation to fit to the purpose of the study. The domains of questions included in the tool were: “socio-demographic characteristics”, “sexual history”, “desire for children”, “family planning awareness”, “family planning use and fertility intentions”, “discussion about FP with health care provider “and perspectives on the quality of FP services”. The questions were translated into Amharic, the local language spoken by respondents, and back translated to English. The final Amharic questionnaire was administered by trained experienced female psychiatric nurses, with an emphasis on a respectful and non- judgmental approach and facilitating the women to be at ease. And the Participants were interviewed after they had completed their follow up visit as an exit interview. Validation was not done in this particular group of participants.

Data quality was controlled by designing a fully structured questionnaire which was pre-tested in five percent of participants in different setup. Interviewers and supervisors were trained for two days. The collected data were examined for completeness and internal consistency each day by supervisors.

### Data analysis

The data were coded and entered using epidata version 3.1 and exported to the Statistical Package for Social Sciences (SPSS) version 20 to be cleaned and analysed. Descriptive statistics were calculated for all variables. In bi-variate analysis, crude odds ratio and confidence intervals were calculated and used to select candidate variables for multivariate analysis using a significance level of p<0.05. Multivariable logistic regression was used to obtain adjusted odds ratios and corresponding 95% confidence interval (CIs). The strength of association was interpreted using the adjusted odds ratio and 95% CI.

### Ethical considerations

Ethical approval was obtained from Saint Paul’s Hospital Millennium Medical College, Institutional Review Board. Informed written consent was obtained from each study participant after informing them about the objectives, risks, and benefits of the study. Participants were informed about their right to participate voluntarily voluntary participation and their right to withdraw from study. We ensured the privacy of the participants during data collection and ensured anonymity of the collected data.

## Results

### Socio-demographic characteristics of respondents

A total of 422 women of reproductive age participated with a response rate of 99.76%. The age distribution of respondents showed that 59% of the participants were in the age group of 18 to 34 years. The mean age of respondents (with one standard deviation) was 32.1 ± 6.7 years. Almost one third of the participants were single (32.93%; n = 139). Four out of ten women were either illiterate or had only primary level education (Table 1).

**Table 1:** Demographic and clinical characteristics of participants (n = 422).

Characteristics	Frequency	percentage
Age in years (mean, standard deviation)	32.06 ± 6.7 (SD)	
<25	63	14.93
25–34	186	44.08
≥35	173	40.99
Marital status		
Single	139	32.94
Married	187	44.31
Widowed	26	6.16
Divorced	70	16.59
Education		
Tertiary level	60	14.22
Secondary level (high school)	195	46.21
Primary level	123	29.14
Illiterate	44	10.43
Diagnosis		
Schizophrenia	170	40.28
Bipolar disorder	116	27.49
Major depressive disorder	136	32.23
Psychotropic medication		
Antipsychotics	208	49.29
Mood stabilizer	67	15.88
Antidepressant	47	34.83
Duration of treatment in months		
1–24	163	38.63
25–48	91	21.56
49 months and above	168	39.81

### Reproductive health characteristics of study participants

Two thirds of the participants (66.1%; n = 279) had a history of pregnancy. Of these women (87.8%; n = 245) had experienced an unintended pregnancy, with (77.6%; n = 190) having an unintended pregnancy for their most recent pregnancy was unintended. More than one third of all respondents, (36.2%; n = 153), have been pregnant at least once after they had been diagnosed as having mental illness. Of these (58.2%; n = 89) pregnancies were unintended and (84.3%; n = 75) had an induced abortion. This is higher compared to only (30.3%; n = 128) of all participants who had a lifetime history of induced abortion. The most common reasons for induced abortion was that the pregnancy was unintended (53.1%; n = 68), the pregnancy arising from forced sexual intercourse (28.9%; n = 37) and fear of the effect of psychotropic medications on the foetus (14%; n = 18). One out of three participants had sexual intercourse before age of eighteen, which can be considered as increased risk of teenage pregnancy (Table 2).

**Table 2:** Reproductive history of participants (N**).

Variable	Frequency	percentage
Age at first time sex (who ever had sex N = 376)		
<18	133	35.37
≥18	243	64.63
Ever forced (N = 376)		
Yes	142	37.77
No	234	62.23
Ever pregnant(N = 376)		
Yes	279	74.20
No	97	25.80
Pregnancy after diagnosis(N = 279)		
Yes	153	54.84
No	126	45.16
Ever had unwanted pregnancy(N = 279)		
Yes	245	87.81
No	34	12.19
Ever had induced abortion(N = 279)		
Yes	128	45.88
No	151	54.12
Total living children (N = 422)		
No children	180	42.65
1–2	158	37.44
3 and above	84	19.91
Desire to have more children in the future(N = 422)		
Yes	169	40.05
No	168	39.81
Not decided yet	85	20.14
Time to have children (N = 169)		
Soon	54	31.95
<1year	31	18.34
1-3years	73	43.19
After 3 years	11	6.52

NB N** N is calculated in this table for each variable

### Family planning related characteristics

Two third (68%; n = 287) of study participants had ever heard about FP methods. The most commonly known methods were the oral contraceptive pill (29.6%; n = 48) the injectable (depot contraceptive) (29%; n = 47), condoms (22.8%; n = 37), contraceptive implant (10%; n = 16), and intrauterine device (5%; n = 8) and six participants (4%) reported knowledge of other FP methods, including natural methods. The most frequently mentioned source of information about FP was health professionals (52.6%; n = 151), a friend or neighbours 20.2% (n = 58), school10.5%(n = 30) and the media 5.2% (n = 22) Types of modern FP methods where information was available were as follows: Pills (30.6%; n = 198), injectable (27.4%; n = 198), intrauterine device (14.7%; n = 95), condoms (14.6%; n = 88) and implants (9%; n = 60) respectively (Table 3).

**Table 3:** Awareness and use of family planning among participants.

Characteristics	Frequency	Percentage
Family planning Awareness (n = 422)		
Yes	287	68.01
No	135	31.99
Do you know Family planning service other than contraceptive *(n = 422)		
Yes	94	22.27
No	328	77.73
Ever used FP methods(n = 422)		
Yes	233	55.21
No	189	44.79
Currently using any FP method(n = 422)		
Yes	162	38.39
No	250	59.24
No Answer	10	2.36
Intention to use (n = 260 for those not using FP currently)		
Intend to use	99	38.08
Not intend to use	156	6.00
Not decided	5	1.92

*Preconception counseling, preventive screening such as cervical cancer and HIV screening and safe abortion.

### Utilization of modern family planning methods

Just over half (56.6%; n = 239) reported that they had ever used FP methods with (38.4%; n = 162) currently using at least one method of FP. The most frequently used method of contraception were pills (29.7%; n = 50) and injectables (26.7%; n = 45). On the other hand, (22%; n = 37), (11.3%; n = 19), and (0.4%; n = 8) of women were using condoms, implant and intrauterine devices respectively. Out of all users, only six of participants used traditional methods with modern contraceptive intrauterine devices or tubal ligation.

In women who were not currently using FP, (60%; n = 156) did not have any intention to use contraception (38.1%; n = 99) did have an intention to use FP in the future and (1.2%; n = 5) were not sure. The reasons for the non-utilization of family planning methods among women are summarized as follows. The most common reason not to use FP was fear that the psychotropic medication was incompatible with the contraceptives (37.8%; n = 61). The second most common reason was fear of contraceptive side effects (20.5%; n = 32) and thirdly (17.9%; n = 28) fear of stigma associated with using FP services as a person with mental illness. Twenty-five women (16.0%) wanted to get pregnant the rest (6.4%; n = 10) they chose to abstain.

### Factors associated with current utilization of modern contraceptive methods

On bivariate analysis, lower number of children, higher FP awareness, previous use of FP, total number of pregnancies, and were associated with utilization of FP. History of unwanted pregnancy and induced abortion associated with lower utilization of family planning (Table 4). Other sociodemographic characteristics including age, education, marital status, income and occupation was not associated with current family planning utilization.

**Table 4:** Factors associated with family planning utilization among participants.

Characteristics	Family planning utilization		COR (95% CI)	AOR (95%CI)	P value
	Yes N (%)	No N (%)			
Number of children					
0	12(32.4	25(67.6)	3.51(1.89,6.53)	1.16(0.49,2.74)	0.07
1–2	64(40.5)	94(59.5)	2.89(1.54,5.44)	2.05(1.06,3.99) *	**0.03**
3 and above	16(19)	68(81.0)	1	1	
FP Awareness					
Yes	96(33.4)	191(66.6)	1	1	
No	66(48.9)	69(51.1)	0.53(0.35,0.81)	1.21(0.65,2.21)	0.57
Ever used FP methods					
Yes	68(29.2 0	165(70.8)	1	1	
No	94 (49.7)	95(50.3)	2.40(1.61,3.59)	0.5(0.28,91)	0.23
Unwanted pregnancy					
Yes	80(67.3)	165(67.3)	1	1	
No	63(47.7)	69(52.3)	1.8(1.2,2.65)	1.10(0.52,2.10)	0.09
Ever Forced to have sex					
Yes	61(43)	81(57)	1	1	
No	81(34.6)	153(65.4)	0.75(0.51,1.13)	0.81(0.50,1.27)	0.34
Induced abortion					
Yes	34(26.2)	96(73.8)	1	1	
No	109(44.1)	138(55.9)	2.2(1.41,3.5)	1.58(0.87,2.87)	0.14
Intention to have child in the future					
Yes	96(56.8)	73(43.1)	1		
No	66(26.1)	187(73.9)	3.73(2.46,5.64)	**0.31(0.19,0.50)**	**0.00**

On multivariable analysis, women who had one or two children had twice the odds of using FP than those who had more than three children adjusted odds ratio (AOR) = 2.05,95% CI: 1.06, 3.99). Women who do not intend to have children were less likely to use FP (AOR = 0 .31, 95% CI: 0.19, 0.51).

## Discussion

In this study women with mental illness utilized FP less than the general population when compared to estimates from previous studies: 38.3% in this study compared to 55.5% of women of reproductive age using any modern contraceptive methods in a study conducted in Addis Ababa [13]. In this context, more than half (58.1%) of the study participants reported having had unwanted pregnancy, indicating high unmet need for FP.

Several studies in Ethiopia suggest that awareness of FP in the general population may have increased substantially despite the low prevalence of use [13, 22–24].

In this study, 60% of respondents mentioned at least one type of family planning method which is lower than findings from other studies conducted in Ethiopia [12, 25]. The reasons for the lower levels of FP coverage in women with mental illness were not explored in the study, but may be explained by poorer access to sexual and reproductive health service and lower educational level of participants.

In the general population in Ethiopia, the order of frequency of methods known and used are injectables, implant, pills, intrauterine device and pills. This is slightly different from this study in which the pill and injectables were the most frequently reported methods [26]. In the current study, awareness about long acting reversible methods including norplant and intrauterine device was very low which is consistent with findings from previous studies carried out in Addis Ababa and a northern regional city [27, 28] but differs from findings from study carried out in a southern regional town [27]. Differences in the sociodemographic, sample size, and study design may have contributed to these differences.

Although declining, traditional methods for family planning are still important, particularly in developing countries such as Ethiopia. However, they were only mentioned by a few participants. This is consistent with a recent report in national survey in which only 1% of participants reported use of these methods [14].

In this study, the common sources of information about FP accessed by women were a health facility, friends or neighbors whereas the mass media was a common source in other studies. Nevertheless, the list is consistent with a recent study conducted in Addis Ababa where 70% of the information sources were from a health care facility [25]. This might be because the participants in the study were recruited from health facility.

In this study current use of FP was 38.5% of married and 38.3% of sexually active un married women. This finding is higher than for married and lower than sexually active unmarried community study in Ethiopia which reported 36% and 58% respectively [13]. The current study finding is also lower than estimated reported a study carried out in Addis Ababa (55% -62%) [23] and higher than a community study from south central Ethiopia findings 25.4% [16, 29].

As the gap between knowledge and use of FP is a measure of unmet need, hence efforts at improving awareness will eventually improve the level of use [29]. The study findings suggest that much more efforts will have to be made to improve awareness about FP in women with mental illness of this group. Only 68% of the women who participated in our study had ever heard about FP methods. This finding is lower than the estimated of 96.5% reported by other studies in the country [30]. Therefore, improving the awareness in this group of women may help to promote FP utilization.

The most common reasons that are known to be responsible for non-use of family planning in this study included stigma, fear of the side effects of methods, myths about family planning and fears of drug -drug interaction. The findings of this study are similar to a study carried out in Nigeria except that spousal opposition was prominent in the Nigerian studies [10, 31, 32]. These reasons pose a serious challenge for routine FP services if the provider has limited knowledge about mental health.

The frequent use of hormonal contraceptive injectable and pills reported in this study contrasts with their third position in the national report [14]. Long-term contraceptive methods were utilized less frequently by participants, which is similar to reports by other studies from Ethiopia and other African countries [26, 33]. Only a few participants reported use of condoms as a contraceptive. The male condom requires the motivation of the sexual partner to use and transfers the burden of fertility regulation from the woman to the male partner. Condoms may be less effective for FP than other methods but offer the benefit of lowering the risk of STDs in high-risk women like those with severe mental illness [24, 25]. The need to educate the male partner on the appropriate use and limitations of condoms should be emphasized, particularly what to do in cases of inappropriate use to avoid unintended pregnancies [17]. Although small number of women in our study participants knew sterilization, none used it conforming to the national trend; sterilization is a poorly accepted or used method in Ethiopia [27, 28, 34]. There is a lot of work that needs to be done to motivate women to use the more effective means of contraception. Women who are motivated to use family planning should be encouraged to use effective methods. In the case of our study participants, discrepancies between awareness and use when compared to the general population may have resulted from the potential hindrances reported by the participants that the regular FP service outlets are unappealing or inaccessible to these patients.

Some of the barriers may be related to stigma, lack of knowledge about psychotropic medication and contraceptive interactions and other illness-related issues. Factors related to the partner were not mentioned as the reason for low FP use; this contrasts with community-based studies in Ethiopia and other similar settings [13, 28, 35].

The other reason could be that women with severe mental illness have frequent contact with mental health workers and low level of interaction with the general health service as reported in a Nigerian study. There is consensus of the need for an individualized approach for advising women with mental illness on FP methods, since inappropriateness may be personalized on the basis of certain problems. For example, the probability of inconsistency of use, deficient cooperation, or poor hygiene may negate use of an intrauterine device and the risk or the presence of depression may discourage the use of the hormonal contraceptives [6].

On the other hand, when compliance cannot be guaranteed and there are no contraindications then injectables can be used [36, 37]. It has been suggested that all patients should be given equal opportunity to use at least a method based on merits. When the patient is indecisive about all methods, then condoms are suggested; in fact, some experts have suggested that it should be used irrespective of any method used because of the protection provided against STDs [37].

The most common reason for using FP in Ethiopia is for limiting birth rather than spacing of births. This contrasts with the reason from Ghana where spacing was the main reason followed by prevention [9]. In this study, the majority of the women had no desire for future children. A substantial number of participants using reversible contraceptive methods, these methods were not compatible with their desire to limit the number of children. This study highlights the importance of specific FP education in health facilities for this community rather than providing general awareness creation only.

According to this study having fewer children was associated with FP service utilization which is in contrary to the study done in Addis Ababa [23]. In this study history of induced abortion was not associated with FP utilization which is consistent with the study from southern Ethiopia [22, 38]. The other factor inversely associated with FP utilization was intention to have children in the future which is consistent with other studies in Addis Ababa [13].

Factors that were shown to be associated with FP utilization in previous studies in Ethiopia such as, age, education, marital status, income and occupation of women were not associated with FP utilization in this study [17, 18, 30, 38]. This may indicate that factors that affect FP utilization in such a vulnerable population as women with mental illness could be different from the general population.

### Limitations

There are some limitations of this study. The study did not obtain information about FP from the male partners. As it is a cross-sectional study it could be difficult to establish cause and effect relationship between the variables. The study was conducted in hospitals where clients thought to have better access for information so the findings may not be generalizable to women with mental illness who do not attend Psychiatric facilities.

## Conclusions

In conclusion, despite its limitations, this study is the first of its kind in Ethiopia to investigate FP among women with severe mental illness in a Hospital setting. Most women with mental illness were not using FP methods. There was low awareness of the FP methods among women with mental illness attending psychiatric outpatient clinics. Family planning education and counselling on family planning for women attending psychiatric care should be strengthened.

## Supporting information

S1 File (DOCX)

S2 File (DOC)

S1 Data (SAV)
