# Supplementary material for: Family planning awareness, utilization and associated factors among women of reproductive age attending psychiatric outpatient care, a cross- sectional study, Addis Ababa, Ethiopia
Source: PLoS One. 2020 Sep 4;15(9):e0238766. doi: 10.1371/journal.pone.0238766 (PMC7473540; doi:10.1371/journal.pone.0238766)
Supplement: S1 File — (DOCX) [file pone.0238766.s001.docx]

| **General Questions**   1. Code Number______________________________ 2. Date of interview ______/_____/_______   (Day/Month/Year) | | | | |
| --- | --- | --- | --- | --- |
| 1. **Interviewer ID............................................** | | | | |
| 1. Result of the interview    1. Complete    2. Incomplete    3. Refused    4. Others | | | | |
| 1. Checked by Investigator: Signature _________________ Date: _____/_____/_________   (Day/ Month/ Year) | | | | |
| **Interviewer** | **Interviewer**: **INTRODUCE YOURSELF TO THE CLIENT** | | |  |
|  | Hello, My name is ..............................................… We are conducting a study to improve the availability and quality of family planning services for psychiatry clients. As part of this, I would like to ask you some questions about the services you have received. There is no risk if you agree to participate in the interview. All the information that you give to me will be kept strictly confidential; your name will not be used and you will not be identified in any way. Your current and future care at this facility will not be affected in any way. This interview should take approximately 30 minutes to complete. Your participation is absolutely voluntary and there is no penalty for refusing to take part. You are free to ask any questions; you may refuse to take part in the interview; you may refuse to answer any question in the interview; and you may stop the interview at any point.  Do you have any questions for me at this time about this survey?  Yes_____ No___  Do you agree to participate in this interview? Yes_____ No_  **IF NO, THANK THE PARTICIPANT AND CLOSE THE INTERVIEW.** | | |  |
|  | _______ _______ _______  Interviewer’s Signature  (Indicates Respondent’s willingness to participate) | | Date:  __/__/_ |  |
| **Interviewer** | **PART I. Socio-demographic characteristics** | | SKIP TO |  |
| 1.1 | How old are you? | ________Years  (age in completed years) |  |  |
| 1.2 | What is your religion? | 1. Orthodox 2. Catholic 3. Muslim 4. Protestant 5. Other (specify) |  |  |
| 1.3 | What is the highest educational level you completed? | 1. Tertiary education 2. High school 3. Primary education 4. Able to read and Write--------- 5. Unable to read & Write ------- 6. No response ---------- 7. Other specify --------- |  |  |
| 1.4 | What ethnic group do you belong to? | 1. Oromo ------------ 2. Amhara----------- 3. Somali---------- 4. Tigre ----------- 5. Gurage ------------ 6. Other (specify) ------ |  |  |
| 1.5 | What is your current marital / relationship status? | 1. Married/cohabited ------ 2. Single, never married ---------- 3. Widowed ------------ 4. Divorced ------------ 5. Non married partner ----- 6. No response ------------ |  |  |
| 1.6 | What is your total monthly income? | 1. Your own income----------- Eth.Birr 2. Husband’s income---------- Eth.Birr 3. Other income sources ____ Eth.Birr 4. No income ------------ 5. Don’t know her own income 6. Don’t know her partner income 7. No response ----------- 8. Other (specify)---------- |  |  |
| 1.7 | What is your current occupation? | 1. Unemployed ------------ 2. Student ------------ 3. Housewife ------------ 4. House servant --------- 5. Daily laborer ------------ 6. Merchant ------------ 7. Government employee 8. Private employee ------- 9. Other (specify)---------- |  |  |
| 1.8 | How long ago did you know that you know you have a psychiatric illness? | Years --------- and  Months ------ |  |  |
| 1.9 | For how long were having treatment and follow up at psychiatric clinic | Years --------- and  Months ------ |  |  |
| 1.9. | What is your specific diagnosis?  (May be copied from the medical record) |  |  |  |
| 1.10 | Have you started taking any medication? | 1. Yes--------- 2. No------ | If No Skip to 1.12 |  |
| 1.11 | If you have started taking medications, what are the medications you are taking?  (May be copied from the medical record) |  |  |  |
|  | **Part II. Sexual history** | |  |  |
|  | Have you ever had sexual intercourse? | 1. Yes--------- 2. No------ 3. No response----------- | Skip to 3.6 |  |
|  | If yes, at what age did you have sex for the first time? | 1. __________ Years old 2. Don’t remember------- 3. No response-------- |  |  |
|  | Was your first sexual experience forced or you didn’t want it | - - 1. Yes     2. No     3. No response |  |  |
|  | Have you ever had sexual intercourse without your will? | 1. Yes  2. No  3. No response |  |  |
|  | Do you have a history of STIs? | 1. Yes……. 2. No ……. 3. Don’t Know 4. No response………. |  |  |
|  | **PART III. Child desire information** | |  |  |
|  | Have you ever been pregnant? | 1. Yes --------- 2. No----------- | skip to 3.8 |  |
|  | What is the total number of pregnancies did you have in the past? |  |  |  |
|  | How many of the pregnancies were after you was diagnosed to have a psychiatric problem? |  |  |  |
|  | Was your last pregnancy wanted/planned? | 1. Yes ------------- 2. No -------------- 3. Don’t know -------- 4. No response --------- |  |  |
|  | Have you ever given birth? | 1. Yes--------- 2. No---------- | Skip to 3.8 |  |
|  | How long ago was your last delivery? | ---------Years and -----------months ago |  |  |
|  | How many biological children do you have? | 1. Living __________ 2. Dead______________ |  |  |
|  | Would you like to have children, or more children, in the future? | 1. Yes ------------- 2. No -------------- 3. Don’t know -------- 4. No response --------- 5. Other (specify) ------- | Skip to 4.1  Skip to 4.1  Skip to 4.1 |  |
|  | If the answer for Q 3.8 is yes, how long would you like to wait before becoming pregnant, or becoming pregnant again? | 1. Number of months:_____ 2. Number of years:______ 3. Other:______ |  |  |
|  | If the answer for Q 3.8 yes, how many (more) children would you like to have in the future? | 1. No. of children desired -------- 2. Don’t know ----------- 3. No response --------- 4. Other (specify) ------- |  |  |
|  | If the answer for Q 3.8 is no, why do you want not to have a child in the future? | 1. Fear of inability to provide care for the unborn baby 2. Fear of side effect of antipsychotic drugs to the unborn baby 3. Have enough family size 4. No response 5. Other (specify) ------- |  |  |
|  | Have you ever had an unwanted pregnancy? | 1. Yes…….. 2. No ……. 3. No response………. |  |  |
|  | Have you ever had an induced abortion? | 1. Yes---------------------- 2. No----------------------- 3. No response---------- | If No Skip to 4.1 |  |
|  | If yes, how many times? | ____________ |  |  |
|  | If yes to question yes 3.13 what was the reason? | - - 1. It was unplanned pregnancy     2. Fear of teratogenicity from antipsychotic drugs     3. The pregnancy resulted from rape/sexual assault     4. Other(specify) __________________ |  |  |
|  | **PART IV. Family planning knowledge** | |  |  |
|  | Did you know any methods of contraception? | 1. Yes ----------- 2. No ----------- | **Skip to 5.2.** |  |
|  | If yes, mention the methods you know | 1. ----------- 2. ----------- 3. ----------- 4. ----------- 5. ----------- 6. ----------- 7. ----------- 8. ----------- 9. ----------- 10. ----------- |  |  |
|  | From where did you get the information regarding the contraception methods | - - 1. Friends     2. School     3. Family member     4. Health care provider     5. Mass media     6. Others specify _________________ |  |  |
|  | Have you ever known other family planning service in addition to the contraception? | 1.Yes  2.No |  |  |
|  | **PART V. Family planning use and fertility intentions** | |  |  |
|  | Have you (or your partner) ever used a family planning method before? | 1. Yes ----------- 2. No ----------- 3. Don’t remember ------ 4. Don’t know --------- 5. No response ---------- 6. Other (specify) -------- | Skip to 4.5  Skip to 4.5  Skip to 4.5  Skip to 4.5 |  |
|  | If yes for Q5.1 specify the method, you /your partner used?  (More than one answer can be possible.) | 1. Condom ----------- 2. Pill (OCP) ----------- 3. Injectable ------------ 4. IUD ---------- 5. Implants --------- 6. Tubal ligation /Vasectomy ----- 7. Breastfeeding ………… 8. Withdrawal method…….. 9. Rhythm method………… 10. No response -------------- 11. Other (specify) ------------ |  |  |
|  | Are you/your partner/ using a family planning method currently (during the study period)? | 1. Yes ----------------- 2. No ---------------- 3. I don’t know ---------- 4. No response ------- | Skip to 4.5  Skip to 4.5 |  |
|  | If yes for question 5.3, specify the method you are using?  (More than one answer can be possible) | 1. Condom ----------- 2. Pill (OCP) ----------- 3. Injectable ------------ 4. IUD ---------- 5. Implants --------- 6. Tubal ligation /Vasectomy --- 7. Breastfeeding ……………… 8. Withdrawal method……………….. 9. Rhythm method……………….. 10. No response --------- 11. Other (specify) --------- | After all responses, skip to 4.8 |  |
|  | If not yes for question 5.3, would you like to use a family planning method in the future? | 1. Yes ----------- 2. No ------------ 3. Don’t know ---------- 4. No response --------- 5. Other (specify) ------- | Skip to 5.7  Skip to 5.7  Skip to 5.7 |  |
|  | If yes, specify the method you intend to use? (More than one answer can be possible) | 1. Condom ----------- 2. Pill (Ocp) ----------- 3. Injectable ------------ 4. IUD ---------- 5. Implants --------- 6. Tubal ligation /Vasectomy --- 7. Breastfeeding ……… 8. Withdrawal method…….. 9. Rhythm method……. 10. No response --------- 11. Other (specify) ------ | After all responses, skip to 6.1 |  |
|  | If no, why don’t you use family planning? | 1. Want to have a child ------ 2. Fear that family planning drugs may affect my health --- 3. I am abstaining from sex ------ 4. Fear of stigma 5. Fear of interaction with my psychotropic medication 6. No response ------- 7. Other specify---------- |  |  |
|  | **PART VI. Discussion on FP with health care provider** | |  |  |
|  | Were you counseled on family planning in the psychiatry clinic any time during your follow-up? | 1. Yes---------- 2. No------------- 3. No response----------- | Skip to 6.3  Skip to 6.3 |  |
|  | If yes, what type of method(s) were you counseled on? | 1. condom---------- 2. Pills --------------- 3. Injectable-------- 4. Norplant………… 5. IUD……………. 6. implant 7. Others (specify)….. |  |  |
|  | Did you receive any family planning method during your visit to this facility? | 1. Yes___ 2. No____ | Skip to 6.5 |  |
|  | If no to question number 6.3. what is the main reason you did not receive a family planning method today? | 1. Came for information only 2. Desire for fertility 3. Changed my mind 4. Pregnancy suspected 5. Cost 6. Method not available 7. Partner objects to method 8. Doctor said I had contraindications 9. Prefer to use a traditional method (i.e. withdrawal, breastfeeding, periodic abstinence) 10. Currently on a long-acting method (i.e. IUD, implants, or injectables) 11. I was referred to other facility 12. Other: 13. Don’t know | After all responses, skip to 6.9 |  |
|  | If yes to question number 6.3, which family planning method(s) did you receive? | 1. condom---------- 2. Pills --------------- 3. injectables-------- 4. Norplant………… 5. IUD……………. 6. Others (specify)….. |  |  |
|  | Did a health provider explain to you how to use the FP method you received? | 1. Yes 2. No 3. Don’t know |  |  |
|  | Did a health provider describe the possible side-effects or what kind of problems may you experience while using this method? | 1. Yes 2. No 3. Don’t know |  |  |
|  | Did a health provider explain what to do if you experience any problems or side effects? | 1. Yes 2. No 3. Don’t know |  |  |
|  | Did the provider mention any other method more convenient to you? | 1. Yes 2. No 3. Don’t know | Skip to 6.11  Skip to 6.11 |  |
|  | Is (Are) this (these) the method(s) you had originally wanted to use? | 1. Yes -------- 2. No -------- 3. Don’t know -------- | Skip to 5.13  Skip to 5.13 |  |
|  | If no to question number 6.10., why did you not receive the method you want to use? | 1. Cost -------- 2. Method not available ---- 3. Partner objected method -------- 4. Doctor said it was not good -------- 5. Doctor said I had contraindications ---- 6. Other: -------- 7. Don’t know -------- |  |  |
|  | Which method(s) were you using before coming to the facility today? | 1. condom---------- 2. Pills --------------- 3. injectable-------- 4. Norplant………… 5. IUD……………. 6. Others (specify)…. | Do not ask respondent this question if she answered “No” to question 6.3 (on FP use). |  |
|  | Have you ever had a problem with a family planning method, wanted to change methods, or wanted to stop? | 1. Yes 2. No 3. Don’t know | Do not ask respondent this question if she answered “No” to question 6.3 (on FP use).  Skip to 7.1 |  |
|  | What was the main problem you had or the main reason you wanted to change or stop using that family planning method? | 1. Physical side-effects 2. Partner did not like the method 3. Pressure from others 4. Fear of infertility 5. Wanted pregnancy 6. Cost 7. Method 8. unavailable/difficult to obtain 9. Other: 10. Don’t know |  |  |
|  | What are you going to do about FP now? | 1. Change to new method 2. Continue with same method 3. Stop using any method 4. Don’t know | Skip to 7.1  Skip to 7.1  Skip to 7.1 |  |
| 6.16 | Which method(s) will you now use? | 1. condom---------- 2. Pills --------------- 3. injectables-------- 4. Norplant………… 5. IUD……………. 6. Others(specify)….. |  |  |
| 6.17 | Did a health provider explain what to do if you want to get pregnant while you are taking your psychiatric medication? | 1. Yes 2. No 3. Don’t know |  |  |
| 6.18 | Did the provider forced you to use any family planning method? | 1. Yes  2. No  3. Don’t know |  |  |
|  | **Part VII. Client’s feelings about the quality of FP services** | |  |  |
|  | Do you feel that you received the information that you wanted about FP? | 1. Yes 2. No |  |  |
|  | Did you feel that your FP consultation was too short, too long, or about the right amount of time? | 1. Too short 2. Too long 3. About the right amount of time |  |  |
|  | In your opinion, did you have enough privacy during the consultation today? | 1. Yes 2. No |  |  |
|  | Generally, are you satisfied with the family planning counseling & services at this hospital? | 1. Yes………………. 2. No…………………. 3. No response…………… |  |  |
|  | Generally, are you felt that family planning counseling & services at FP clinic ideal for women with mental illness? | 1. Yes………………. 2. No……………… 3. No response……… |  |  |
