# Supplementary material for: Family planning awareness, utilization and associated factors among women of reproductive age attending psychiatric outpatient care, a cross- sectional study, Addis Ababa, Ethiopia
Source: PLoS One. 2020 Sep 4;15(9):e0238766. doi: 10.1371/journal.pone.0238766 (PMC7473540; doi:10.1371/journal.pone.0238766)
Supplement: S2 File — (DOC) [file pone.0238766.s002.doc]

| **ጠቅላላ ጥያቄዎች**   1. መለያ ቁጥር______________________________ 2. መጠይቁ ተደረገበት ቀን______/_____/_______   (ቀን/ ወር/ ዓ.ም.) | | |
| --- | --- | --- |
| 1. የመጠይቁ ሁናቴ    1. የተሟላ    2. ያልተሟላ    3. ተቋውሞ    4. ሌላ | | |
| 1. በተመራማሪውየተረጋገገጠ…………………………………..ፊርማ……………………..ቀን:_____/_____/_____ ( ቀን/ ወር/ ዓ.ም) | | |
| **መጠይቅ አድራጊው** | **ቃለ መጠይቅ አድራጊው እራስዎን ለተሳታፊው ያስተዋውቁ** | |
|  | ጤና ይስጥልኝ ስሜ…………………………………………………………………በአእምሮ ህክምና አገልግሎት ተጠቃሚዎች ላይ የቤተሰብ እቅድ አገልግሎት አጠቃቀም ላይ ምርምር እያደረግን ነው፡፡ስለሆነም ስለአገኙት አገልግሎት አንዳነድ መጠይቆችን አደርግልዎታለሁ፡፡u²=I Ø“ƒ uSd}õ­ U”U ›Ã’ƒ Ñ<Çƒ ›Ã•`U ::u²=I Ø“ƒ ¨pƒ ¾T>Öc<ƒ ማንኛውም መረጃ S[Í T>eØ^©’~ ¾}Öuk ’¨<:: S[Í¨< ¨Å ¢Uú¨<}` ŸÑv u%EL SKÁ eKTÃ•[¨< T>eØ^© ÃJ“M T”—¨<U ¾`f” T”’ƒ ¾T>ÑMê S[Í uêG<õ J” unM ›Ã¨×U::uተቋሙአሁን ወይም ወደፊት በሚያገኙት አገልግሎት ላይ ምንም አይነት ተፅዕኖ አይኖረውም፡፡ ÃI” SÖÃp SS<Lƒ cLd Åmn w‰ ÁeðMÑªM:: u²=I Ø“ƒ ¨<eØ ¾T>d}óƒ S<K< uS<K< u`f ðnÅ˜’ƒ eKJ’ uT”—¨<U ¨pƒ Ø“~” TqU Ã‹LK<:: S[Í ¾cÖ< u=J”U ”"D”  ስለጥናቱ ሊጠይቁኝ የሚፈልጉት ነገር ይኖራልን?  አዎ የለም--------  በጥናቱ ውስጥ ለመሳተፍ ፈቃደኛ ነዎት?  አዎ የለም----------  መልሱ የለም ከሆን ፣አባክዎ አመስግነው ያሰነብቱዋቸው | |
|  | በመጠይቅ አድራጊው የተረጋገገጠ (ለመሳተፍ ፈቃደኝነቱን) ፊርማ ቀን: _____/_____/_________  (ቀን/ ወር/ ዓ.ም) |  |

| በመጠይቅ አድራጊው | **ክፍል አንድ Socio-demographic characteristics** | | SKIP TO |
| --- | --- | --- | --- |
| 1.1 | አድሜዎ ስንት ነው? | ________ አመት በሙሉ ቁጥር ይቀመጥ |  |
| 1.2 | GÃT•ƒ­ U”É” ’¨< | 1. *`„Ê¡e ¡`e+Á” 2. ካ„K=¡ 3. ፐሮቲስታንት 4. S<eK=U 5. K?L ÃÑKî |  |
| 1.3 | ከፍተኛ ¾ƒUI`ƒ Å[Í­ | 1. ሶሰሰተኛ ደረጃ(ከሃይስኩል በላይ) ሁለተኛ ደረጃ /ሃይስኩል 2. የመጀመሪያ ደረጃ 3. ማነበብ እና መፃፍ መቻል 4. ማነበብ እና መፃፍ ያለመቻል 5. መልስ የለም 6. አላውቅም |  |
| 1.4 | የጋብቻ ሁናቴ | 1. ÁÑu< 2. ¾}KÁ¿ /¾}ó~ 3. vKu?†¨< ¾V}v†¨< 4. ÁLÑu< 5. ›w[¨< ¾T>•\ (IÒ© ÁMJ’ Òw‰) 6. መልስ አልተሰጠም |  |
| 1.5 | አጠቃላይ የወር ገቢዎ ምን ያህል ነው? | 1. የራስዎ ገቢ-------------------ብር 2. የባለቤትዎ ገቢ-------------------ብር 3. ሌላ የገቢ ምንጭ 4. የራሴን ገቢ አላውቅም 5. የባለቤቴን ገቢ አላውቅም 6. ገቢ የለንም--------- 7. መልስ የለም ----------- 8. ሌላ(ይገለፅ)------------- |  |
| 1.6 | የስራ ሁኔታ? | 1. ስራ የሌለው 2. ተማሪ 3. የቤት እመቤት 4. የቤት ሰራተኛ 5. የቀን ሰራተኛ 6. ነጋዴ 7. የመንግስት ተቀጣሪ የግል ድርጅት ሰራተኛ 8. ሌላ (ይገለጽ) |  |
| 1.7 | የአእምሮ ህመም እነዳለብዎ ያወቁት መቼ ነው? | ______ አመት እና--------------ወሮች | በወራት ቁጥር ይቀመጥ |
| 1.8 | ለምን ያህል ጊዚ በአእምሮ ህመ ምህክምናና ክትትል ቆይተዋል | ___ወሮች | በወራት ቁጥር ይቀመጥ |
| 1.8 | ህመምዎምንድነው(ከካርድሊታይይችላል)? | ---------------------------------- |  |
| 1.9 | መድሃኒትመውሰድጀምረዋል? | 1. አዎ--------- 2. የለም------ | If No Skip to 2.1 |
| 1.10 | መድሃኒት እየወሰዱ ከሆነ ፣የሚወሰወዷቸው መድሀኒቶች ምን ምን ናቸው?(ከካርድ ሊታይ ይችላል)? | -  ------------------------------------------- |  |
|  | **Part II. Sexual history** | |  |
|  | ወሲብ አደርገው ያውቃሉ? | 1. አዎ--------- 2. የለም ------ 3. መልስ የለም ----------- | Skip to 3.6 |
|  | መልስዎ አዎ ከሆነ በየትኛው እድሜዎ ለላ ነው ለመጀመሪያ ጊዜ ያደረጉት? | 1. __________ አመቴ ላይ 2. አላስታውሰውም 3. መልስ የለም ----------- |  |
|  | የመጀመሪያ የወሲብ ግንኙነትዎ በግዳጅ ውይም ሳይፈልጉ የሆነ ነበር? | 1. አዎ--------- 2. የለም ------ 3. መልስ የለም ----------- |  |
|  | ከፍላጎትዎ ውጪ ወሲብ አድርገው ያውቃሉ? | 1. አዎ--------- 2. የለም ------ 3. መልስ የለም ----------- |  |
|  | የአባላዘር በሽታ ዘዎት ያውቃል? | 1. አዎ--------- 2. የለም ------ 3. አላውቅም 4. መልስ የለም ----------- |  |
|  | **PART III. Child desire information** | |  |
|  | አርግዘው ሆነው ያውቃሉ? | 1. አዎ--------- 2. የለም ------ | skip to 3.8 |
|  | በአጠቃላይ ምነ ያህል እረግዝና ነበርዎ/ስነት ጊዜ አርግዘው ነበር? | ------------------ |  |
|  | ምን ያህል እረግዝናዎቹ ናቸው አእምሮ ህመምዎን ካወቁ በኋላ የነበሩት ? | --------------- |  |
|  | የመጨረሻው እረግዝናዎ /የታቀደ /የተፈለገ ነበር? | 1. አዎ--------- 2. የለም ------ 3. መልስ የለም ----------- |  |
|  | ልጅ ወልደው ያውቃሉ ? | 1. አዎ--------- 2. የለም ------ | Skip to 3.8 |
|  | ለመጨረሻ ጊዜ የወለዱት መቼ ነበር? | --------- አመታት እና -----------ወሮች በፊት በፊት |  |
|  | ስነት ልጆች አሉዎት/ወልደዋል? | 1. በህይወት ያሉ 2. በህይወት የሌሉ __________ |  |
|  | ወደፊት ልጅ መውለድ ወይም ተጨማሪ ልጆች ማግኘት ይፈልጋሉ? | 1. አዎ--------- 2. የለም ------ 3. አላውቅም 4. መልስ የለም ----------- | Skip to 3.11  Skip to 4.1  Skip to 4.1 |
|  | ለጥያቄ 3.8. መልስዎ አዎ ከሆነ ምንያህል ግዜ እረጉዝ ሳይሆኑ መቆየት ይፈልጋሉ/ድጋሚ እርጉዝ? | 1. ----------- ወሮች 2. -------------አመታት 3. ሌላ:______ |  |
|  | ለጥያቄ 3.8. መልስዎ አዎ ከሆነ ምንያህል ልጆችን ወደፊት መውለድ ፈልጋሉ? | 1. ------ያህልልጆች 2. አላውቅም 3. መልስ የለም 4. ሌለ(ይገለፅ) |  |
|  | ለጥያቄ 3.8. መልስዎ የለም ከሆነ ለምን ልጅ መውለድ አይፈልጉም? | 1. የተወለደውን ልጅ መንከባክብ አልችልም ብሎ መፍራት 2. የመድሀኒቶችን ጎንዮሽ ውጤት ለህፃኑ መፍራት 3. በቂ የቤተሰብ ቁጥር አለኝ 4. መልስ የለም 5. ሌለ(ይገለፅ) |  |
|  | ያልተፈለገ እረግዝና ኖርዎት ያውቃል | 1. አዎ--------- 2. የለም ------ 3. መልስ የለም ----------- |  |
|  | እረግዝናአሰወርደው ያውቃሉ? | 1. አዎ--------- 2. የለም ------ 3. መልስ የለም ----------- | If No Skip to 4.1 |
|  | አዎ ከሆነ ስነት ጊዜ? | ____________ |  |
|  | ለጥያቄ 3.13 አዎከሆነምክነያትዎምንነበር? | - - 1. እርግዝናው ያልታቀደ ነበር     2. ከመዳኒቱ የሚመጣውን ጉዳት በመፍራትs     3. እረግዝናው የተፈጠረው በግዳጅ /ያለፈቃድ በተፈፀመ ወሲብ ነው     4. ሌላ(ይገለፅ)   __________________ |  |
|  | **PART IV. Family planning knowledge** | |  |
|  | የወሊድ መከላከያ መንገዶችን ያውቃሉ? | 1. አዎ ----------- 2. የለም ----------- | **Skip to 5.1** |
|  | አዎ ከሆነ መልሱ እባከዎ ይጥቀሱልኝ | 1. ----------- 2. ----------- 3. ----------- 4. ----------- 5. ----------- 6. ----------- 7. ----------- 8. ----------- 9. ----------- 10. ----------- 11. ---------- |  |
| 4.3. | ስለወሊድ መከላከያ መረጃ ከየት አገኙ | - - 1. ከጓደኛ     2. ከትምህረት ቤት     3. ከቤተሰብ አባል     4. ከጤና ተቋም     5. ከመገናኛ ብዙሃን     6. ሌላ(ይገለፅ)   __________________ |  |
| 4.4. | ከወሊድ መቆጣጠሪያ በተጨማሪ የምታውቂው የቤተሰብ እቅድ አገልግሎት አለ ? | 1. አዎ ----------- 2. የለም ----------- |  |
|  | **PART V. Family planning use and fertility intentions** | |  |
| 5.1. | እርስዎ ወይም የትዳር አጋርዎ የቤተሰብ እቅድ አገለግሎት ተጠቅመው ያውቃሉ? | 1. አዎ ----------- 2. የለም ----------- 3. አላስታውስም | Skip to 5.5  Skip to 5.5 |
| 5.2. | መልስዎ ለጥያቄ 5.1 አዎ ከሆነ እባክዎ ዘዴውን /አይነቱንይነገሩን (ከአንድ መልስ በላይ መስጠት ይቻላለል? | 1. ኮንዶም 2. እንክብል/ኪነን 3. መርፌ 4. ሉፕ/በማህጸን የሚቀመጥ 5. ኢምፐላነት /በክንድ የሚቀበር 6. የማህጸፀን ቱቦ ማሰቋጠር/ማከላሸት 7. ጡት ማጥባት 8. ማቋረጥ/የዘርፍሬን ከውጪ ማፍሰስ 9. ቀን ቆጥሮ የመጠቀም ዘዴ 10. መልስ የለም 11. ሌላ(ይገለፅ) |  |
| 5.3. | እርስዎ ወይም የትዳር አጋርዎ የቤተሰብ እቅድ አገለግሎት አሁን በመጠቀም ላይ ናችሁ?(በጥናቱ ወቅት) | 1. አዎ ----------- 2. የለም ----------- 3. አላስታውስም 4. መልስ የለም ------- | Skip to 5.5  Skip to 5.5 |
| 5.4. | መልስዎ ለጥያቄ 5.3 አዎ ከሆነ እባክዎ ዘዴውን /አይነቱንይነገሩን (ከአንድ መልስ በላይ መስጠት ይቻላለል? | 1. ኮንዶም 2. እንክብል/ኪነን 3. መርፌ 4. ሉፕ/በማህጸን የሚቀመጥ 5. ኢምፐላነት /በክንድ የሚቀበር 6. የማህጸፀን ቱቦ ማሰቋጠር/ማከላሸት 7. ጡት ማጥባት 8. ማቋረጥ/የዘርፍሬን ከውጪ ማፍሰስ 9. ቀን ቆጥሮ የመጠቀም ዘዴ 10. መልስ የለም 11. ሌላ(ይገለፅ) |  |
| 5.5. | መልስዎ ለጥያቄ 5.3 አዎ ካልሆነ ወደፊት የቤተሰብ እቅድ አገልግሎት መጠቀም ያስባሉ? | 1. አዎ ----------- 2. የለም ----------- 3. አላውቅም | Skip to 5.7  Skip to 5.7 |
| 5.6. | መልስዎ ለጥያቄ 5.5አዎከሆነእባክዎየሚመርጡትን ዘዴውን /አይነቱን ይነገሩን (ከአንድመልስበላይመስጠትይቻላለል? | 1. ኮንዶም 2. እንክብል/ኪነን 3. መርፌ 4. ሉፕ/በማህጸንየሚቀመጥ 5. ኢምፐላነት /በክንድየሚቀበር 6. የማህጸፀን ቱቦ ማሰቋጠር/ማከላሸት 7. ጡት ማጥባት 8. ማቋረጥ/የዘር ፍሬን ከውጪ ማፍሰስ 9. ቀን ቆጥሮ የመጠቀም ዘዴ 10. መልስ የለም 11. ሌላ(ይገለፅ) | After all responses, skip to 6.1 |
| 5.7. | መልስዎ ለጥያቄ የለም ከሆነ ለምን መጠቀም እነደማይፈልጉ ምክነያትዎን ይንገሩኝ? | 1. ልጅ ለማግኘት ስለምፈልግ 2. የወሊድ መቆጣጠሪያ መድሀኒቶች ጤናዬን ይጎዳሉ ብዬ ስለማስብ 3. ከወሲብ በተአቅቦ ስላለሁ 4. መገለልን ስለምፈራ 5. መድሀኒቶቹ ከአእምሮ ህክምና መድሀኒቶች ጋራ ይጋጫል ብዬ ስለምፈራ 6. መልስ የለም 7. ሌላ (ይገለፅ) |  |
|  | **PART VI. Discussion on FP with health care provider** | |  |
| 6.1. | በአእምሮ ህክምና ክፍል ውስጥ ስለቤተሰብ እቅድ አገልግሎት ተነግርዎት/ምክር ተሰጥተዎ ያውቃል? | 1. አዎ ----------- 2. የለም ----------- 3. አላስታውስም | Skip to 6.3  Skip to 6.3 |
| 6.2. | አዎ ከሆነ መልሰዎ በየትኛው ዘዴ ነው ተነግርዎት የሚያውቀው? | 1. ኮንዶም 2. እንክብል/ኪነን 3. መርፌ 4. ሉፕ/በማህጸንየሚቀመጥ 5. ኢምፐላነት /በክንድየሚቀበር 6. ሌላ ይገለጽ |  |
| 6.3. | በዚ ህህክምና ተቋም የቤተሰብ እቅድ አገልግሎት ተጠቅመዋል? | 1. አዎ ----------- 2. የለም ----------- | Skip to 6.5 |
| 6.4. | ለጥያቄ ቁጥር 6.3 መልስዎ የለም ከሆነ ዋናው ምክንያትዎ ምንድነው? | 1. የመጣሁት መረጃ ልጠይቅ ብቻ ነው 2. ልጅ መውለድ ፈልጋለሁ 3. ሀሳቤን ቀይሬ ነው 4. እርግዝና ሊኖረኝ ስለሚችል 5. ዋጋው 6. የምፈልገው ዘዴ አልነበረም 7. የትዳር አጋሬ ተቃወመ 8. ዶክተሩ መውሰድ የማልችልበት ሁኔት እነዳለብኝ ነገረኝ 9. ባህላዊ ዘዴን(ጡት ማጥባት፡ዘርን ውጬ ማፍሰስ፣ቀን ቆጥሮ በመጠቀም 10. በአሁኑ ወቅት ለረጀም ጊዜ የሚቆይ ዘዴ እየተጠቀምኩ ነው(ሉፕ፣በከንድየሚቀበረውን፣መርፌ) 11. ሌላ የጤና ተቋም ፈልጌ 12. ሌላ 13. አላውቅም | After all responses, skip to 6.9 |
| 6.5. | ለጥያቄ 6.3 መልስዎአዎን ከሆነ ተጠቅመው የሚያውቁት ዘዴ ዬትኛው ነው? | 1. ኮንዶም 2. እንክብል/ኪነን 3. መርፌ 4. ሉፕ/በማህጸንየሚቀመጥ 5. በክንድ የሚቀበር 6. ሌላ(ይገለፅ) |  |
| 6.6. | የቤተሰብ እቅድ አገልግሎትን እንዴት እነደሚጠቀሙ የጤና ባለሙያው ገለፃ አድርገዎለሎት ነበር | 1. አዎ 2. የለም 3. አላውቅም |  |
| 6.7. | የቤተሰብ እቅድ አገልግሎት ሲጠቀሙ ምን አይነት የጎንዮሽ ውጤት ወይም ጉዳት ሊገጥሞት እነደ ሚችል የጤና ባለሙያው ገለፃ አድርጎልዎታል | 1. አዎ 2. የለም 3. አላውቅም |  |
| 6.8. | የቤተሰብ እቅድ አገልግሎት ሲጠቀሙ የጎንዮሽ ውጤት ወይም ጉዳት ቢገጥሞት ምን ማድረግ እነዳለብዎ የጤና ባለሙያው ገለፃ አድርጎልዎታል? | 1. አዎ 2. የለም 3. አላውቅም |  |
| 6.9. | የጤና ባለሙያው ለእርስዎ ይበልጥ ምቹ የሆነ ዘዴ ጠቁምዎታል? | 1. አዎ 2. የለም 3. አላውቅም | Skip to 6.11  Skip to 6.11 |
| 6.10. | ዘዴው/ዘዴዎቹ እርስዎ የመረጡት /የመረጧቸው ናቸውን? | 1. አዎ 2. የለም 3. አላውቅም | Skip to 5.13  Skip to 5.13 |
| 6.11. | ለጥያቄ 6.10 መልስዎ የለም ከሆነ ለምነድነው የመረጡትን ዘዴ/ዘዴዎች ያልተጠቀሙት? | 1. በዋጋው ምክንያት 2. የምፈልገው ዘዴ አልነበረም 3. የትዳር አጋሬ ተቃወመ 4. ዶክተሩ ጥሩ አልነበረም 5. ዶክተሩመውሰድ የማልችልበትሁኔታ እነዳለብኝ ነገረኝ 6. ሌላ(ይገለጽ) 7. አላውቅም |  |
| 6.12. | ወደተቋሙ ከመምጣትዎ በፊት ይጠቀሙ የነበሩት ዘዴ ምንድነው? | 1. ኮንዶም 2. እንክብል/ኪነን 3. መርፌ 4. ሉፕ/በማህጸንየሚቀመጥ 5. በክንድ የሚቀበር 6. ሌላ(ይገለፅ) | Do not ask respondent this question if she answered “No” to question 6.3 (on FP use). |
| 6.13. | እየተጠቀሙ ያሉትን የቤተሰብ እቅድ አገልግሎትን ለመቀየር ወይም ለማቆም ተቸግረው ወይም ችግር ገጥምዎት ያውቃል? | 1. አዎ 2. የለም 3. አላውቅም | Do not ask respondent this question if she answered “No” to question 6.3 (on FP use).  Skip to 7.1 |
| 6.14. | እየተጠቀሙ ያሉትን የቤተሰብ እቅድ አገልግሎትን ለመቀየር ወይም ለማቆም ምን አይነት ችግር ገጥምዎት ያውቃል? | 1. አካለዊ የጎነዮሽ ውጤት 2. የሌሎች ግፊት 3. መካነንትን ፍራቻ 4. እረግዝና ፈልጌ 5. ዋጋው 6. የምፈልገው ዘዴ አልነበረም ወይም የለም 7. ሌላ 8. አላውቅም |  |
| 6.15. | አሁን ስለቤተሰብ እቅድ ምን ሊያደርጉ አስበዋል? | 1. ዘዴውን ልቀይር 2. እየተጠቀምኩት ያለሁትን ዘዴ ልቀጥል 3. መጠቀም ላቆም 4. አላውቅም | Skip to 7.1  Skip to 7.1  Skip to 7.1 |
| 6.16 | የትኛውን ዘዴ ሊጠሙ አሰቡ? | 1. ኮንዶም 2. እንክብል/ኪነን 3. መርፌ 4. ሉፕ/በማህጸንየሚቀመጥ 5. በክንድ የሚቀበር 6. ሌላ(ይገለፅ) |  |
| 6.17 | የአእምሮ ህክምና መድሃኒት እየወሰዱ ማርገዝ ቢፈልጉ ምን ማድረግ እነዳለብዎ የጤና ባለሙያው ነግርዎታል? | 1. አዎ 2. የለም 3. አላውቅም |  |
| 6.18 | የጤና ባለሙያው የቤተሰብ እቅድ አገልግሎት እነዲ ጠቀሙ አስገደድዎት ያውቃልን? | 1. አዎ 2. የለም 3. አላውቅም |  |
|  | **Part VII. Client’s feelings about the quality of FP services** | |  |
|  | ስለቤተሰብ እቅድ አገልግሎት የሚፈልጉትን መረጃ እነዳገኙ ይሰማዎታልን | 1. አዎ 2. የለም |  |
|  | የተሰጥዎት የምክር ጊዜ በጣም ያጠረ፣በጣም የረዘመ ወይም ተመጣጣኝ ነው ብለው ያስባሉ? | 1. በጣም አጭር 2. በጣም ረጅም 3. ጠመጣጣኝ |  |
|  | በእርስዎ አመለካከት አገልግሎቱን በሚያገኙበት ወቅት በቂ ነፃነት አግኝተዋል/በበቂ ሁኔታ በግል ነበር የተጠየቁት | 1. አዎን 2. የለም |  |
|  | በአጠቃላይ እዚህ ሆስፒታል ስለሚሰጠው የቤተሰብ እቅድ አገልግሎት እና ምክር ረክተዋል? | 1. አዎን 2. የለም 3. መልስ የለም |  |
|  | በአጠቃላይ አሁን ያለው የቤተሰብ እቅድ እገልግሎት የአእምሮ ህመም ላለባት ሴት ምቹ እና ተመጣጣኝ ነው ብለው ያስባሉን? | 1. አዎን 2. የለም 3. መልስ የለም |  |

| **የመጠይቅ አድራጊው አስተያየት-----------------------------------------------------------------------------------------------------------------------------------------------------**  **እባከዎ ተሳታፊውን ስለ ተሳትፎአቸው እና ስለ ጊዜአቸው አመስግነው ያሰናብቱዋቸው** |
| --- |
